# Perception of Lebanese Obstetricians Toward COVID-19 Vaccination During Pregnancy and Breastfeeding

**DOI:** 10.7759/cureus.105035

**Published:** 2026-03-11

**Authors:** Hassan Hamze, Elie Hobeika, Usta M Ihab, Sami Taghlabi, Christelle Dagher, Anwar Nassar

**Affiliations:** 1 Obstetrics and Gynecology, American University of Beirut Medical Center, Beirut, LBN; 2 Pediatrics, Baylor College of Medicine, Houston, USA

**Keywords:** covid-19, obstetricians, perception, pregnancy, vaccine

## Abstract

Introduction: In response to the drastic complications that resulted from Coronavirus-19 (COVID-19) infection, vaccines were developed to contain its rapid spread. However, concerns were raised regarding the safety of COVID-19 vaccines for pregnant and breastfeeding women. This study aimed to explore the perceptions and practices of Lebanese obstetricians regarding the COVID-19 vaccine, highlighting the barriers to recommending it.

Methods: A cross-sectional study was implemented using a questionnaire. Descriptive statistics in the form of frequencies and percentages were utilized to get an overview of obstetricians’ characteristics and responses. Inferential statistics were employed to examine plausible barriers to recommending the vaccine.

Results: A total of 265 obstetricians were included in the study. Most of the physicians had received the vaccine themselves (254 (95.8%)) and were recommending the vaccine to all of their pregnant patients (238 (89.8%)). Lack of data about the safety of the vaccine and that of clear guidelines were the main reasons for not recommending it to all patients. Receiving the vaccine, having sufficient data about the vaccine, adequate knowledge about COVID-19 infection and vaccination, the type of vaccine, and managing patients who were exposed to COVID-19 were significant predictors of offering the vaccine.

Conclusions: Our results provided an overview of Lebanese obstetricians’ attitudes and practices regarding the COVID-19 vaccine and identified some of the factors that hinder the recommendation of the vaccine. Such information might be helpful to overcome the obstacles that prevent obstetricians from abiding by international recommendations, thus increasing the uptake of vaccines by pregnant women in any potential future pandemic.

## Introduction

Since the diagnosis of the first case of Coronavirus-19 (COVID-19) in December 2019, more than 778 million cases have been identified, with a death toll that exceeded seven million individuals globally [1]. The virus primarily affects the pulmonary system, leading to critical symptoms and multisystem complications [2]. Hence, multiple efforts, even in the early phase of the pandemic, were directed toward developing a safe vaccine that would be capable of protecting populations worldwide against COVID-19 infection. The very first COVID-19 vaccine was introduced on July 22, 2020 [1,3]. In Lebanon, the vaccine received Emergency Use Authorization (EUA) in December 2020, but the first vaccination campaign commenced on February 14, 2021 [4]. To date, more than 13.6 billion vaccine doses have been administered globally [1]. However, in the case of pregnant and breastfeeding women, contradicting opinions emerged regarding their vaccinations in the initial phase of the pandemic. In light of the fact that this population was regarded as a vulnerable group and was thus excluded in the very first trials conducted to assess the safety of the vaccines [5], some specialists have argued that pregnant women should not be vaccinated. Others have encouraged vaccinating this high-risk population, knowing that the course of COVID-19 in pregnancy might be more complicated and associated with maternal and fetal threats [6].

Vaccines are known to be among the most effective ways of preventing infectious diseases, but safety data were not available for obstetricians. A recent systematic review and meta-analysis explored safety concerns regarding COVID-19 vaccination for pregnant women. No specific adverse pregnancy outcomes linked to vaccine uptake were reported [7], whereas another study documented a beneficial effect, demonstrating that maternal and fetal morbidity and mortality, including preterm birth, are decreased in association with receiving one or two doses of the vaccine [8]. An observational study determining the immunogenicity to COVID-19 among pregnant women who had received the vaccine suggested that immunity is well established in both mother and child [9], with the triggered humoral immune response causing side effects that are similar to those in non-pregnant women [8].

The American College of Obstetricians and Gynecologists (ACOG) recommended the vaccine early during the pandemic to all pregnant and lactating women with no contraindications to receive it. Moreover, they advised moderately to severely immunocompromised patients to receive a third dose of the Pfizer-BioNTech or Moderna mRNA COVID-19 vaccine [10]. Unlike their initial recommendations of vaccinating only high-risk pregnant women, the Royal College of Obstetricians and Gynecologists (RCOG) now recommends that all pregnant women receive the vaccine [6]. Similarly, the Royal Australian and New Zealand College of Obstetricians and Gynecologists (RANZCOG) recommended the Pfizer mRNA vaccine routinely to pregnant women [11,12]. After ACOG, the International Federation of Gynecology and Obstetrics (FIGO) released a statement on March 2, 2021, supporting the COVID-19 vaccine for pregnant women [13].

Despite these recommendations from international medical boards, the perceptions and attitudes of women toward receiving the COVID-19 vaccination were variable [14,15], which were mainly based on the recommendation of their obstetricians [16]. Thus, it is beneficial to examine the knowledge and attitudes of obstetricians regarding COVID-19 vaccination. Within the Lebanese context, only one study examined the knowledge, attitudes, and practices of Lebanese obstetricians toward COVID-19 infection and pregnancy, with no focus on the vaccine [17]. Subsequently, the aim of our study was to address the literature gap by investigating the attitudes and practices of Lebanese obstetricians pertaining to receiving the COVID-19 vaccination by pregnant and breastfeeding women, and to assess barriers toward offering the vaccine.

## Materials and methods

Study design and setting 

This study employed a national cross-sectional design conducted among Lebanese obstetricians and gynecologists.

Eligibility criteria

Participants were considered eligible for recruitment if they met the following inclusion criteria: (1) certified obstetrician and gynecologist (OBGYN), (2) of Lebanese nationality, and (3) affiliated with the Lebanese Order of Physicians (LOP) and registered in the Lebanese Society of Obstetrics and Gynecology (LSOG).

Recruitment of participants

After receiving the ethical approval from the institutional review board (IRB) at the American University of Beirut Medical Center (AUBMC), the names and contact information of registered obstetricians were obtained from the LOP on March 29, 2022. Survey distribution started on April 25, 2022. Eligible obstetricians received an official email containing a (1) cover letter explaining the purpose of the study and its expected benefits, (2) a consent form, and (3) the questionnaire, in English language, utilized to collect the data. A reminder email was sent to all participants within four weeks of the initial email, reminding non-responders.

Data collection

The questionnaire is composed of four sections (Appendix). The first section collected demographic data including gender, age, graduating school, type of practice, years in practice, and any subspecialty. We gathered information on whether the participants had personally received the vaccine or not, along with their reasons for declining it. The second section solicited practice patterns of COVID-19 vaccination for pregnant women. The third section stressed the knowledge regarding COVID-19 infection in pregnancy, its symptoms, and association with serious morbidity, in addition to physicians’ willingness to prescribe the COVID-19 vaccine if it was available across Lebanon and the reasons for their reservations about the vaccine. The last section assessed their views toward giving a vaccine to previously infected women, or high-risk patients, their perceptions toward different types of vaccines, and management strategies adopted for infected women.

Statistical analysis

Both descriptive and inferential types of statistics were conducted. For the former type of statistics and given that all questionnaire items are of a categorical nature, frequencies and percentages were mainly utilized to describe the sample population and provide an overview of the responses collected, highlighting the common perceptions, attitudes, and practices of obstetricians regarding COVID-19 vaccination. Furthermore, inferential statistics were implemented in the form of a chi-squared test or Fisher’s exact test, depending on whether the sample sizes support the approximation, to explore any associations between questionnaire items, including obstetricians’ recommendations of the COVID-19 vaccine and demographic data. Advanced multivariate analysis was executed in the form of an ordinal logistic regression model, given the ordered nature of the dependent variable (recommending the COVID-19 vaccine). A p-value < 0.05 was considered statistically significant. All statistical analyses were carried out using the SPSS package version 25 (IBM Corp., Armonk, NY).

Ethical considerations

A consent form was attached to the initial email sent to eligible obstetricians. It emphasized the following major points: (1) study’s main objectives, (2) any potential risks or benefits that might result from participating in the study, (3) participants’ right to withdraw from the study at any point or to skip any question that could bring them discomfort, (4) confidentiality and anonymity of participants’ personal information, and (5) preservation of participants’ data securely within all the research team exclusively. If consent was secured, participants were asked to fill out all the survey questions.

## Results

Demographic properties of the sample population

Out of 1,090 obstetricians registered with the LSOG and LOP, 148 were retired, had left the country, did not work in obstetrics, had wrong e-mails, or were not available. A total of 265 eligible obstetricians responded, with a response rate of 28.1%. Most of the obstetricians were males (176 (66.4%)), had their medical OBGYN training in Lebanon (188 (70.9%)), were in private practice (182 (68.7%)), and had more than 10 years of experience practicing OBGYN (191 (72.1%)). Only 38 (14.3%) obstetricians had subspecialties, and given the small number, our findings were not compared based on this variable (Table 1).

**Table 1:** Characteristics of sample population *Presented as mean (standard deviation)

Variables	Categories	Frequency (n)	Percentage (%)
Gender	Male	176	66.4
	Female	89	33.6
	Total	265	100.0
Country of graduation	Lebanon	188	70.9
	Dual with Lebanon	3	1.1
	Others	74	27.9
	Total	265	100.0
Type of practice	Private practice	182	68.7
	University-affiliated medical center	64	24.2
	Government-affiliated medical center	19	7.2
	Total	265	100.0
Years of experience	<5 years	30	11.3
	5-10 years	44	16.6
	>10 years	191	72.1
	Total	265	100.0
Subspecialty	Yes	38	14.3
	No	227	85.7
	Total	265	100.0
Year of graduation*	1994.42 (119.844)		

Obstetricians’ attitudes, knowledge, and practices regarding the COVID-19 vaccine

A significant majority of the respondents had received the COVID-19 vaccine themselves (254 (95.8%)), with the exception of 11 (4.2%) who did not. Table 2 summarizes obstetricians’ attitudes toward COVID-19 vaccination. Among those who did not receive the vaccine, seven (63.6%) chose so because they do not receive recommended vaccines, and three (27.3%) had doubts about the safety of the vaccine. As for recommending the COVID-19 vaccine to pregnant or breastfeeding women, 238 (89.8%) of obstetricians recommended it to all patients, while 24 (9.1%) recommended the vaccine only for high-risk patients, mostly due to a lack of safety data and potentially unidentified long-term effects on both the baby and the mother (15 (55.6%)).

**Table 2:** Overview of participants’ responses COVID-19: Coronavirus-19

Questionnaire items	Categories	Frequency (n)	Percentage (%)
Received COVID-19 vaccine	Yes	254	95.8
	No	11	4.2
	Total	265	100.0
Reason for taking COVID-19 vaccine	Fear of contracting COVID-19 infection	24	9.4
	Recommended for all healthcare professionals	45	17.7
	My age	13	5.1
	Others	1	0.4
	Multiple reasons	171	67.3
	Total	265	100.0
Reason for not taking COVID-19 vaccine	I do not usually take vaccines	7	63.6
	I am allergic to eggs	1	9.1
	I have doubts about the safety of the vaccine	3	27.3
	Total	11	100.0
Vaccine was recommended for pregnant or breastfeeding patients?	Yes, for all patients	238	89.8
	Yes, for high-risk patients	24	9.1
	No	3	1.1
	Total	265	100.0
Reason for not recommending COVID-19 vaccine for all patients?	There are no clear guidelines that it should be given to all pregnant women	11	40.7
	There is not enough data about its safety and long-term effects on baby and mother	15	55.6
	Other	1	3.7
	Total	27	100.0
Duration between an infection and offering the vaccine to obstetric patient?	After 1 month	14	5.9
	After 3 months	200	84.0
	After pregnancy	20	8.4
	Not at all	4	1.7
	Total	238	100.0
As a physician, considering self as having sufficient data and information about COVID-19 vaccine to adequately counsel pregnant and breastfeeding women	Yes	227	85.7
	No	38	14.3
	Total	265	100.0
Source of information	Media	4	1.5
	International societies	55	20.8
	Medical journals	4	1.5
	Ministry of Health	2	0.8
	Others	1	0.4
	Multiple sources	199	75.1
	Total	265	100.0
Consider pregnant or breastfeeding women at a higher risk of developing complications after COVID-19 compared with non-pregnant individuals	Yes	257	97.0
	No	8	3.0
	Total	265	100.0
If vaccine was available at the clinic or in the pharmacies, will physician recommend COVID-19 vaccine to all healthy obstetric patients?	Most likely not	5	1.9
	Definitely yes	114	43.0
	Most likely yes	123	46.4
	Not sure	23	8.7
	Total	265	100.0
If yes or most likely yes, trimester for recommending the vaccine	Any trimester	207	87.3
	Only 2nd trimester and above	17	7.2
	During the first antenatal visit	10	4.2
	Based on the risks	3	1.3
	Total	237	100.0
Decision affected by type of vaccine	Yes	149	56.2
	No	116	43.8
	Total	265	100.0
Managed pregnant women who were exposed to a direct contact with documented COVID-19?	Yes	139	52.5
	No	126	47.5
	Total	265	100.0
If yes, treatment given	Vitamins	10	7.2
	Aspirin	2	1.4
	Panadol	6	4.3
	Others	1	0.7
	Multiple medications	120	86.3
	Total	139	100.0
Would you offer your patients to receive the 3rd dose of a vaccine?	Yes	238	89.8
	No	27	10.2
	Total	265	100.0

The majority of 227 (85.7%) obstetricians believed that they had sufficient information about COVID-19 vaccine to adequately counsel pregnant and breastfeeding women, and 199 (75.1%) relied on multiple sources to receive the necessary information about the vaccine encompassing media, international societies, medical journals, and the Ministry of Health, whereas 55 (20.8%) relied solely on international societies. Two hundred fifty-seven (97%) believed that pregnant women were at a higher risk of developing complications compared with non-pregnant individuals.

If the vaccine was available at the clinic or in pharmacies, 23 (8.7%) were not sure if they would offer it, and five (1.9%) would most likely refrain. The majority of those who offered the vaccine would recommend it at any trimester (207 (87.3%)), whereas 17 (7.2%) would recommend it in the second trimester or beyond. The type of the vaccine would affect the decision of 149 (56.2%) on whether or not to offer the vaccine. Moreover, 139 (52.5%) reported that they managed women who contracted COVID-19, with 120 (86.3%) utilizing multiple medications including aspirin, Panadol, vitamins, ivermectin, and low-molecular-weight heparin (LMWH). Regarding offering a third dose of the vaccine, 238 (89.8%) affirmed that they would offer it to their patients (Table 2).

Predictors of recommending the COVID-19 vaccine to pregnant or breastfeeding women

None of the characteristics attributed to the participating obstetricians has significantly affected their decisions to recommend the COVID-19 vaccine (Table 3). Factors that were significantly different (p-value < 0.05) between obstetricians who offered the COVID-19 vaccine and those who did not are summarized in Table 4. Most of the obstetricians who got vaccinated had recommended the vaccine to all patients (235 (92.5%)), while most of the unvaccinated obstetricians had recommended the vaccine to only high-risk patients (5 (45.5%)) (p-value < 0.001; χ^2^ = 73.078). The majority of those who believed they had sufficient information about the vaccine did recommend it to all patients (223 (98.2%)), whereas 20 (52.6%), among those who had insufficient information, recommended the vaccine to only high-risk patients (p-value < 0.001; χ^2^ = 117.116). Across obstetricians who believed that pregnant women are at a higher risk of developing complications than non-pregnant ones, 233 (90.7%) recommended the vaccine to all patients, whereas only 22 (8.6%) and two (0.7%) recommended the vaccine to only high-risk patients and no patients, respectively (p-value = 0.016; χ^2^ = 8.569).

**Table 3:** Differences in recommending the COVID-19 vaccine based on participants’ characteristics Odds ratio could not be generated due to the categorical nature of variables (>2 groups) COVID-19: Coronavirus-19

Variables		Do you recommend COVID-19 vaccine to your pregnant or breastfeeding patients? (n (%))			p-value (chi-squared value (*χ*^2^))
		Yes, for all patients	Yes, for high-risk patients	No	
Gender	Male	156 (88.6%)	18 (10.2%)	2 (1.1%)	0.287 (0.590)
	Female	82 (92.1%)	6 (6.7%)	1 (1.1%)	
Age	<30 years	14 (100.0%)	0 (0.0%)	0 (0.0%)	0.103 (1.735)
	30-35 years	27 (93.1%)	1 (3.4%)	1 (3.4%)	
	36-40 years	39 (88.6%)	4 (9.1%)	1 (2.3%)	
	41-45 years	42 (95.5%)	2 (4.5%)	0 (0.0%)	
	46-50 years	39 (88.6%)	5 (11.4%)	0 (0.0%)	
	>50 years	77 (85.6%)	12 (13.3%)	1 (1.1%)	
Country of graduation	Lebanon	171 (91.0%)	16 (8.5%)	1 (0.5%)	0.119 (1.813)
	Dual with Lebanon	3 (100.0%)	0 (0.0%)	0 (0.0%)	
	Others	64 (86.5%)	8 (10.8%)	2 (2.7%)	
Type of practice	Private practice	162 (89.0%)	17 (9.3%)	3 (1.6%)	0.198 (1.011)
	University-affiliated medical center	58 (90.6%)	6 (9.4%)	0 (0.0%)	
	Government-affiliated medical center	18 (94.7%)	1 (5.3%)	0 (0.0%)	
Years in practice	<5 years	28 (93.3%)	1 (3.3%)	1 (3.3%)	0.508 (0.003)
	5-10 years	39 (88.6%)	4 (9.1%)	1 (2.3%)	
	>10 years	171 (89.5%)	19 (9.9%)	1 (0.5%)	
Subspecialty	Yes	37 (97.4%)	1 (2.6%)	0 (0.0%)	0.067 (2.712)
	No	201 (88.5%)	23 (10.1%)	3 (1.3%)	

**Table 4:** Association between recommending COVID-19 and other questionnaire items Odds ratio could not be generated due to the categorical nature of variables (>2 groups) ᵃSignificant p-value < 0.05. COVID-19: Coronavirus-19

Questionnaire items		Do you recommend COVID-19 vaccine to your pregnant or breastfeeding patients? (n (%))			p-value (chi-squared value (*χ*^2^))
		Yes, for all patients	Yes, for high-risk patients	No	
Received COVID-19 vaccine	Yes	235 (92.5%)	19 (7.5%)	0 (0.0%)	0.000 (73.078)ᵃ
	No	3 (27.3%)	5 (45.5%)	3 (27.3%)	
As a physician, considering self as having sufficient data and information about COVID-19 vaccine to adequately counsel pregnant and breastfeeding women	Yes	223 (98.2%)	4 (1.8%)	0 (0.0%)	0.000 (117.116)ᵃ
	No	15 (39.5%)	20 (52.6%)	3 (7.9%)	
Consider pregnant or breastfeeding women at a higher risk of developing complications after COVID-19 compared with non-pregnant individuals	Yes	233 (90.7%)	22 (8.6%)	2 (0.7%)	0.016 (8.569)ᵃ
	No	5 (62.5%)	2 (25.0%)	1 (12.5%)	
Decision affected by type of vaccine	Yes	145 (97.3%)	4 (2.7%)	0 (0.0%)	0.000 (20.557)ᵃ
	No	93 (80.2%)	20 (17.2%)	3 (2.6%)	
Consider COVID-19 vaccine teratogenic or harmful when given in pregnancy?	Yes	1 (11.1%)	8 (88.9%)	0 (0.0%)	0.000 (45.389)ᵃ
	No	237 (92.6%)	16 (6.3%)	3 (1.2%)	
Managed pregnant women who were exposed to a direct contact with documented COVID-19?	Yes	135 (97.1%)	4 (2.9%)	0 (0.0%)	0.000 (16.874)ᵃ
	No	103 (81.7%)	20 (15.9%)	3 (2.4%)	

Most of the respondents who believed that the type of vaccine impacted their decision had recommended it to all patients (145 (97.3%)), in comparison to four (2.7%) who recommended it to only high-risk patients (p-value < 0.001; χ^2^ = 20.557). A majority of eight (88.9%) out of nine obstetricians who believed that the COVID-19 vaccine is teratogenic had recommended the vaccine to only high-risk patients, in contrast to one (11.1%) who recommended the vaccine to all patients (p-value < 0.001; χ^2^ = 45.389). Among obstetricians who managed pregnant women exposed to COVID-19, 135 (97.1%) have recommended the vaccine to all patients, yet only four (2.9%) recommended it to high-risk patients (p-value < 0.001; χ^2^ = 16.874).

Referring to Table 5, it can be noted that the created ordinal regression model explains 59.1% of the variability of recommending the COVID-19 vaccine (p-value < 0.001). Only three independent factors showed significance in the model. With every unit increase in receiving the COVID-19 vaccine, having sufficient information about it, and managing contracted patients (moving up from yes to no), there is a 0.077 (p-value = 0.020; B = -2.560 (95% CI (-4.712, -0.408))), 0.017 (p-value < 0.001; B = -4.033 (95% CI (-5.530, -2.536))), and 0.111 (p-value = 0.006; B = -2.193 (95% CI (-3.765, -0.622))) times less likely chance for recommending COVID-19 vaccine to decrease to a lower unit (moving down from no to yes, only to high-risk patients, then to yes, for all patients).

**Table 5:** Advanced regression model for recommending the COVID-19 vaccine Reference group: recommended vaccine = 3 (no). Significance of final model = 0.000. McFadden pseudo R-squared = 0.591. ᵃYes, for all patients. ᵇYes, for high-risk patients. ᶜSignificant p-value < 0.05. COVID-19: Coronavirus-19

Parameters		Estimate (B)	Exp. (B)	Standard error	Significance	95% CI lower bound	95% CI upper bound
Threshold	Recommended vaccine = 1ᵃ	-4.541	-	1.670	0.007ᶜ	-7.814	-1.268
	Recommended vaccine = 2ᵇ	0.606	-	1.261	0.631	-1.865	3.078
Location	Received COVID-19 vaccine = 1ᵃ	-2.560	0.077	1.098	0.020ᶜ	-4.712	-0.408
	Having sufficient information = 1ᵃ	-4.033	0.017	0.764	0.000ᶜ	-5.530	-2.536
	Pregnant women more at risk = 1ᵃ	-0.745	0.474	1.142	0.514	-2.983	1.493
	Type of vaccine affects decision = 1ᵃ	-1.427	0.240	0.749	0.057	-2.894	0.041
	Is COVID-19 teratogenic = 1ᵃ	1.297	3.658	1.023	0.205	-0.709	3.303
	Manage contracted‎ patients = 1ᵃ	-2.193	0.111	0.802	0.006ᶜ	-3.765	-0.622

## Discussion

Most of the Lebanese obstetricians, who took part in the survey, recommended the COVID-19 vaccine to all pregnant or breastfeeding mothers (238 (89.8%)), with a small proportion recommending it only to high-risk patients. Lack of sufficient data about the safety of the vaccine was a major contributor to hesitancy toward offering the COVID-19 vaccine. Overall, obstetricians were knowledgeable about COVID-19 infection and the vaccine. Moreover, the obstetricians’ uptake of the vaccine, having sufficient information about the vaccine, COVID-19 infection and vaccine knowledge, type of the vaccine, and disease management were significant predictors of recommending the COVID-19 vaccine to all patients.

There is limited literature exploring obstetricians’ perceptions and attitudes toward the COVID-19 vaccine. One study examined perceptions and knowledge of obstetricians and midwives concerning the COVID-19 vaccine, and it was reported that 65 (78.3%) obstetricians had taken at least one dose of the COVID-19 vaccine [18], which is lower than the 254 (95.8%) reported in this study. Ours is closer to that reported in another observational study, which included 16 obstetricians and midwives (16 (100%)) [19]. Among all healthcare professionals included, 34 (71%) would recommend COVID-19 to their patients [19], which is lower than that reported in our study (238 (89.8%)).

Interestingly, in a cross-sectional study that examined perceptions regarding the COVID-19 vaccine of healthcare workers encompassing obstetricians, it was found that the rate of recommending the vaccine increased from 12% to 49% after the recommendation of vaccinating pregnant women was officially published [20]. A review probed some of the barriers that stand in the way of receiving the COVID-19 vaccine among patients and healthcare providers. Lack of data pertaining to the safety of the vaccine and concerns about plausible teratogenicity to both the mother and fetus were the major concerns highlighted [21], which are similar to the barriers underscored in our study. In comparison to studies that explored perceptions and attitudes of healthcare professionals toward other vaccines like the influenza vaccine, common barriers to recommending vaccination included insufficient evidence regarding its efficacy and limited or a lack of knowledge regarding the vaccine [22-24]. Other distinct barriers entailed anti-vaccination campaigns, misconceptions, and lack of access to the vaccine [23-25].

Despite the fact that the majority of obstetricians in our sample had received the COVID-19 vaccine, a small proportion refrained from getting vaccinated. The major reason behind not taking it is attributed to usually not taking vaccines, which definitely calls for further speculation underlying such behavior, for better management. Among obstetricians not recommending the vaccine to all patients, they reasoned it to the lack of safety data and clear guidelines for the vaccine. Not receiving the COVID-19 vaccine, insufficient data about the vaccine, inadequate knowledge about COVID-19 infection, regardless of the type of vaccine, belief that the vaccine is teratogenic, and not managing patients who were exposed to COVID-19 were all significant predictors of either recommending the vaccine to only high-risk patients or not recommending it at all.

Given that the study mainly relied on a questionnaire to collect the necessary data, non-response bias and recall bias could have affected the results reported. Moreover, due to the limited number of research studies that have addressed a research problem similar to ours, a thorough comparison with the literature was not possible. In light of the time gap present since the execution of this study, our findings might not reflect current clinical practice due to the increased evidence in what concerns COVID-19 vaccination during pregnancy. To the best of our knowledge, our study is the first to be conducted in Lebanon and the Middle East and North Africa (MENA) region, exploring attitudes and practices of obstetricians regarding the COVID-19 vaccine. It included obstetricians from various areas across Lebanon, which aids in generalizing our results at the national level, despite the low response rate, because, while comparing the demographic data of the included obstetricians in comparison to all registered obstetricians, the differences were found to be insignificant. Furthermore, our results highlighted the reasons behind some obstetricians’ reluctance to recommend the vaccine, which is an under-reported issue with an impact that goes beyond COVID-19 itself.

## Conclusions

The findings portrayed in this study enrich the literature and provide an overview of obstetricians’ opinions and their practices in recommending the COVID-19 vaccine, while underscoring significant barriers. Such information is vital to understanding obstacles that may hinder offering the COVID-19 vaccine, allowing the application of suitable measures, including regular obligatory awareness campaigns and workshops for obstetricians to attend in order to keep them updated with any arising medical issues and concerns related to their field of practice. The information provided in our study can assist in applying the appropriate measures to increase the rate of recommending vaccination among obstetricians, thus increasing the rate of vaccination uptake among pregnant and breastfeeding women in preparation for future pandemics.

## Appendices

The COVID-19 Vaccine Questionnaire-Physician Survey

Serial Number: __________________           Date of interview: ___________________

Please choose the single best answer, except when asked to give more than one option.

1. Gender: □ Male  □ Female

2. Age: □ <30 years  □ 30-35 years  □ 36-40 years  □ 41-45 years  □ 46-50 years  □ >50 years

3. OB/GYN degree obtained in (year) ____________ from (country) ________________

4. Practice: □ Private practice  □ University-affiliated medical center  □ Government-affiliated medical center

5. Years in practice: □ <5 years  □ 5-10 years  □ >10 years

6. Any subspecialty?  □ No  □ Yes

7. If the answer to question 6 is Yes, what subspecialty? □ Maternal-fetal medicine  □ Oncology  □ Reproductive endocrinology  □ Urogynecology

8. Do you practice? □ Only OBS  □ Only GYN  □ Both OBS & GYN

9. Did you receive the COVID-19 vaccine? □ Yes  □ No

If the answer to question 9 is No, skip to question 11

10. Why did you take the COVID-19 vaccine? (You can choose more than one answer) (Then skip to question 12)

□ I usually take any available vaccine

□ Because of the fear of contracting COVID-19 infection

□ Because it is recommended for all healthcare professionals

□ Because of my age

□ Others (specify): ______________________________________________

11. Why did you choose not to take it? (You can choose more than one answer)

□ My name did not come up

□ I do not usually take vaccines

□ I am allergic to eggs

□ I have medical problems

□ Others (specify): ______________________________________________

12. Do you recommend the COVID-19 vaccine to your pregnant patients? □ Yes  □ No

If the answer to question 12 is Yes, skip to question 14

13. Why don’t you recommend the COVID-19 vaccine for your patients?

□ There are no clear guidelines that it should be given to all pregnant women

□ There is not enough data about its safety and long-term effects on the baby and mother

□ Others (specify): ______________________________________________

14. As an obstetrician, do you think you have sufficient data and information about the COVID-19 vaccine in order to adequately counsel pregnant women? □ Yes  □ No

15. Where do you get such information? (You can choose more than one answer)

□ Media (TV, newspapers, …)

□ Medical journals

□ Internet

□ Medical conferences

□ Ministry of Health

□ Others (specify): ______________________________________________

16. What are the symptoms that you inform your patients can occur with COVID-19? (You can choose more than one answer)

□ Fever

□ Cough

□ Sore throat

□ Body aches

□ Vomiting

□ Diarrhea

17. Based on your knowledge, are pregnant women at a higher risk of developing complications after COVID-19 compared with non-pregnant individuals?

□ Yes  □ No  □ Not sure

18. If it was available for the public, would you offer the COVID-19 vaccine routinely to all healthy pregnant women?

a. Definitely not

b. Most likely not

c. Definitely yes

d. Not sure

19. If the answer to question 18 is either a, b, or d (definitely not, most likely not, and not sure), why did you choose that option? (You can choose more than one answer)

a. Fear that it will have an effect on the fetus in utero

b. Fear that it might have a negative effect on the health of the mother

c. Lack of enough data on its safety

d. Lack of enough data on its efficacy

e. Fear of any long-term side effects on the child after birth

f. The cost of the vaccine

g. Other causes: ________________________________________________

20. If the answer to question 18 is c (definitely yes), in which trimester would you recommend it? (You can choose more than one answer)

□ Any trimester

□ Only 2nd trimester and above

□ Only 3rd trimester and above

□ During the first antenatal visit

□ Based on the season

21. Would the type of vaccine affect your decision on whether to give it or not?  □ Yes  □ No

22. Would you recommend the COVID-19 vaccine to exposed pregnant women? □ Yes  □ No  □ Not sure

23. Would you recommend the COVID-19 vaccine to asymptomatic pregnant women with coexistent medical problems? □ Yes  □ No  □ Not sure

If the answer to question 23 is No, skip to question 25

24. Which existing medical problems? (You can choose more than one answer)

□ Asthma

□ Diabetes

□ Hypertension

□ Cardiac disease

□ Others (specify): ______________________________________________

25. Did you manage pregnant women who were exposed to a direct contact with documented COVID-19? □ Yes  □ No

If the answer to question 25 is No, skip to question 28

26. How did you manage them?

a. Supportive therapy

b. Perform PCR multiple times

c. Started vitamins prophylactically

d. Referred them to an infectious disease specialist

27. Did you advise that she should not see her household? □ Yes  □ No

28. Do you believe that COVID-19 is teratogenic when given in the first trimester? □ Yes  □ No

29. Did you manage pregnant women who contracted COVID-19, either by high suspicion of symptoms or by testing positive?

□ Yes  □ No

If the answer to question 29 is No, you are done with the survey. We appreciate your time. Thank you.

30. How did you manage them?

a. Supportive therapy

b. Admitted to the hospital

c. Remdesivir, steroids, or antibiotics at home

d. Referred them to an infectious disease specialist

You are done with the survey. We appreciate your time. Thank you.
